# Precision medicine advancements in glioblastoma: A systematic review

**DOI:** 10.37796/2211-8039.1403

**Published:** 2023-06-01

**Authors:** Karan Iyer, Shubham Saini, Suman Bhadra, Sohini Kulavi, Jaya Bandyopadhyay

**Affiliations:** Department of Biotechnology, Maulana Abul Kalam Azad University of Technology, NH-12 (Old NH-34) Simhat, Haringhata, Nadia, 741249, West Bengal, India

**Keywords:** Blood brain barrier, Brain tumor, Cancer, Precision medicine, Glioblastoma

## Abstract

**Background:**

Glioblastoma multiforme, commonly known as GBM or glioblastoma is a grade IV astrocytoma. Brain tumors are difficult to treat and lead to poor prognosis and survival in patients. Gliomas are categorized into four different grades among which GBM is the worst grade primary brain tumor with a survival of less than a year. The genomic heterogeneity of the brain tumor results in different profiles for patients diagnosed with glioblastoma. Precision medicine focuses on this specific tumor type and suggests specialized treatment for better prognosis and overall survival (OS)

**Purpose:**

With the recent advancements in Genome-Wide Studies (GWS) and various characterizations of brain tumors based on genetic, transcriptomic, proteomic, epigenetic, and metabolomics, this review discusses the advancements and opportunities of precision medicine therapeutics, drugs, and diagnosis methods based on the different profiles of glioblastoma.

**Methods:**

This review has exhaustively surveyed several pieces of works from various literature databases.

**Conclusion:**

It is evident that most primary brain tumors including glioblastoma require specific and precision therapeutics for better prognosis and OS. In present and future, molecular understanding and discovering specific therapies are essential for treatment in the field of neurooncology.

## 1. Introduction

Glioblastoma multiforme (GBM) is the most common primary brain tumor occurring in almost 80% of the primary malignant central nervous system (CNS) tumors. Glioblastoma occurs in about 3 in 100,000 people every year. The prevalence of the tumor is more in Caucasians compared to Asians and Africans. Furthermore, the tumor prevalence is 1.5 times more in men compared to women [1]. The overall survival (OS) of patients diagnosed with glioblastoma is less than 1 year to 14 months approximately [2].

Glioblastoma patients experience pressure in their head region due to the tumor growth and face symptoms like nausea, headache, drowsiness, personality changes and seizures. Cell cycle dysregulation is associated with enhanced glioblastoma cell proliferation [3]. As visualized through MRI (magnetic resonance imaging), most commonly the tumor occurs in the supratentorial (cerebrum) location of the brain including the frontal, temporal, and parietal lobes [4]. Glioblastoma mostly occurs in the frontal lobe, and seldom the tumor progresses in both the occipital and temporal lobes, known as the butterfly glioma [5]. Studies suggest that the emergence of glioblastoma or brain tumors is associated with exposure to ionizing radiation. Few theories suggest the cause of the tumor as a consequence of long-term mobile phone use. However, present evidences still require further confirmation and reliable data [6]. It has a high invasive ability and malignant form but it does not tend to metastasize to other parts of the human anatomy, apart from the brain and spinal cord [7]. Glioblastoma is a grade IV astrocytoma caused by the glial cells (astrocytes) in the brain and CNS. Astrocytes are one of the four glial cells that has major functions in the brain and spinal cord including their function in axon guidance, synaptic support, and further acting as a blood brain barrier (BBB). These cells are star-shaped brain cells, and thus obtaining their name astrocytes.

The astrocytes are crucial for the formation and maintenance of the BBB. The BBB, being a highly selective membrane, has a significant role in the therapeutics of glioblastoma as the complex epithelial-like tight junctions within the brain endothelium does not permit certain solutes to pass through [3]. The BBB is also regarded as a blockade as many anti-cancer drugs administered (oral and intravenous) gets restricted and is not readily penetrable. The ability of the drugs to penetrate the BBB are dependent on pharmacokinetic properties and its formulation. Low molecular size, weight and increased lipophilicity of the drugs are characteristics enabling easy permeability.

One certain incapable drug is Imatinib [8], imatinib mesylate (a tyrosine kinase inhibitor) for targeting cancer cells, which is regarded as the “Magical bullet” for the treatment of chronic myeloid lymphoma (CML) [9]. Imatinib, also known as “Gleevac” has been recorded as one of the first cancer therapies showing potential with a targeted action in CML in early 1990s. Unfortunately, Imatinib has low efficiency in targeting brain tumor cells as it penetrates the BBB poorly [10]. The formulation of Imatinib along with methamphetamine has shown positive signs of BBB penetration for treatment of both CML and glioblastoma. Hence, an understanding of different combined formulations specific for BBB penetration and glioblastoma therapies must be explored and applied for personalized treatment. The current standard of treatment for glioblastoma is non-specific and does not categorize glioblastoma patients based on the genetic profile of the tumor. Developments in the field of GWS and a better understanding of the categorized profiles paves way for specific and personalized medicine for glioblastoma therapy. Personalized combined therapies designing for different varieties of brain tumor profiles are under research and await a breakthrough.

## 2. Glioblastoma: A grade IV astrocytoma

**Fig. 1:**
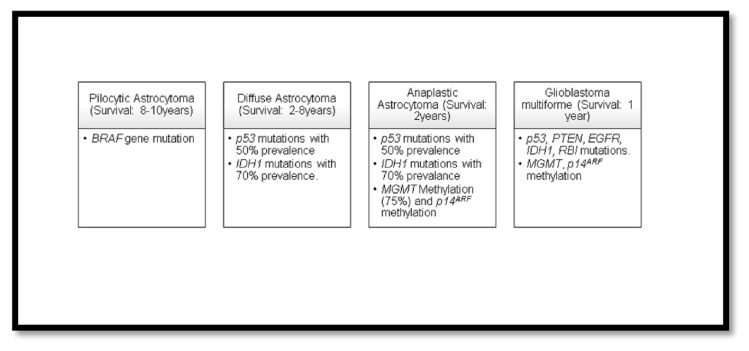
WHO classification of four grades of gliomas and occurrence of mutations (provide reference for this).

Gliomas are categorized into four different grades by the World Health Organization (WHO) on account of their malignancy and survival rates in patients. Glioblastoma (Grade IV) is the most severe one compared to the rest with OS of subjects <1 year. Pilocytic astrocytoma is categorized as grade I with survival period of 8–10 years. Diffuse astrocytoma is categorized as grade II with a survival of 7–8 years after diagnosis. Anaplastic astrocytoma is categorized as grade III with OS of 2–3 years [11]. The different grades of gliomas categorized by the WHO display different mutations. The following data [Fig. 1] provides information on different mutations occurring in the various grades of gliomas.

Glioblastoma is further classified into two types, primary and secondary glioblastoma. The primary glioblastoma is more malignant and has a poor prognosis compared to the secondary. Majority of the primary glioblastoma mutations include those of *EGFR* (epidermal growth factor receptor) amplifications (40%–60%), *p53* (tumor protein P53) mutations (30%), *PTEN* (phosphatase and TENsin homolog) mutations (25%), and the loss of entire chromosome 10 (70%). Most cases of primary glioblastoma tumor involve *MGMT* (O (6)-methylguanine-DNA methyltransferase) methylation at a very low occurrence of 36%. The secondary glioblastoma has mutations in *EGFR* (10% amplification), *p53* (65%), *IDH1* (isocitrate dehydrogenase) (70% mutations), and *MGMT* (methylation 75%). The significance of methylation of the mutated gene plays a major role in the disease prognosis, recurrence, and therapy [12]. The low-grade gliomas such as the grade I (Pilocytic astrocytoma) and II (Diffuse astrocytoma) transform into the secondary glioblastoma at later stages, while the ones which form directly (*de novo*) are known as the primary glioblastoma. Secondary glioblastoma also appears in patients of younger age of 40 when compared to primary glioblastoma, occurring in elderly at an age of 60 [13,14].

### 2.1. Genetic heterogeneity in glioblastoma

Many novel and developing cancer treatments involve the identification of a single genomic alteration and targeting the same for therapy. The same principle may be impractical and unrealistic in glioblastoma. The reason is due to the multiple alterations in its genomic, transcriptomic, and epigenetic profile in glioblastoma. This indicates that in the case of glioblastoma, it undergoes more than a single genetic or cellular event and hence, requiring multiple combinations of therapies to target individual events.

Glioblastoma shows both inter-tumoral and intra-tumoral heterogeneity [13]. Inter-tumoral heterogeneity is the various mutations that occur in different glioblastoma patients. The genetic profile of the glioblastoma tumor differs among patients, thus demanding specific/personalized treatments. Inter-tumoral heterogeneity is further classified into four subtypes classic, neural, pro-neural, and mesenchymal subtypes [14]. The classification of the subtypes is based upon the molecular alterations. The prognosis of patients in mesenchymal subtype is the worst amongst the four subtypes.

**Fig. 2:**
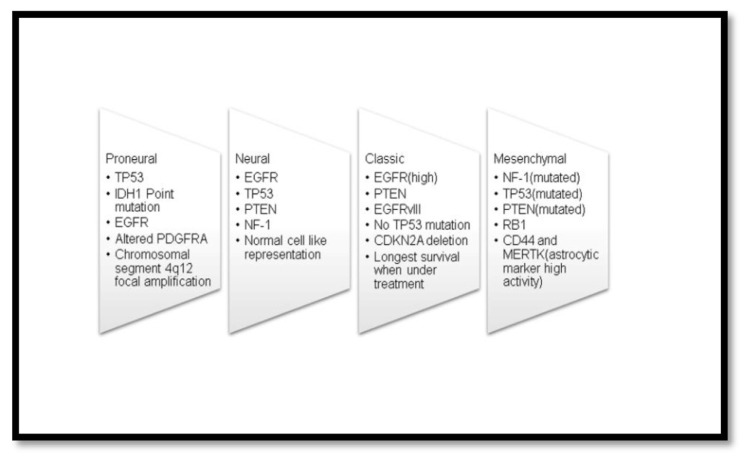
Four subtypes of inter tumor heterogeneity and occurrence of genetic alterations amongst the four subtypes.

Generation of genomic profiles of glioblastoma has been possible with the progression of The Cancer Genome Atlas (TCGA) [15]. Molecular characterization of different subtypes of glioblastoma along with its genetic alterations has major significance in novel therapies [1]. Development of mouse models with molecular alterations in the aforementioned genes [Fig. 2], has been in progress by the employment of genetic deletion systems like the recombinase enzyme Cre-loxP system® [16]. These types of approaches for the development of mouse models are in research to enhance the preclinical trials and track down therapies using specialized anti-cancer agents for glioblastoma. Robust mouse models for each subtype are still under development.

EGFR (epidermal growth factor receptor) activating mutations exist dominantly in the classic subtypes. The mesenchymal subtype, which has the worst prognosis, is often associated with NF-1 and TP53 mutations [17,18]. PDGFR (platelet-derived growth factor receptor) mutation often exists dominantly in the pro-neural subtypes. PDGFR activation and related VEGF (vascular endothelial growth factor) show neovascularization and tumor growth in glioblastoma. Pre-clinical trials using anti-angiogenic agents targeting PDGFR deactivation are under research. Clinical trials demonstrated that the drug Imatinib (Gleevac), was effective against the PDGFR with 6 months progression free survival and median OS of 48.9 weeks [19–21]. Novel drugs are thus desired as Imatinib does not effectively pass through the BBB. Furthermore, several non-coding constraint mutations display regulatory potential by multiple mutations overlapping transcription factor binding sites, and reducing the DNA binding capacity. Hence, these act as additional candidate glioblastoma genes for tumor regulation in glioblastoma [22].

**Fig. 3:**
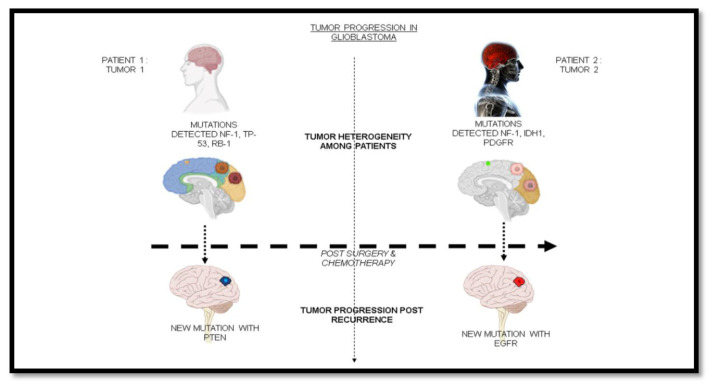
Tumor progression amongst profiles in GBM along with heterogeneity of mutations at different stages of the tumor growth.

Apart from the inter-tumor heterogeneity among patients diagnosed with glioblastoma, glioblastoma tumors display an intra-tumor heterogeneity, i.e. varied mutations existing within the tumor. Hence, targeting a single mutation does not lead to therapy in all cases [Fig. 3]. Multiple combinations of drugs are required for existing intra-tumor heterogeneity. The tumor often arises with mutations leading to signal transduction pathway activation of those downstream of tyrosine kinase receptors, such as EFGR and PDGFR [23]. Evidence shows that the cells carry mutations in PDGFR, EFGR, and Receptor Tyrosine Kinase (RTK), all existing together in glioblastoma [24].

The absence of 1p/19q co-deletion in IDH1 mutations exhibited greater significance in the survival rates of patients [25]. General mutations involved with glioblastoma are MGMT, IDH1, TP53, RB1, RTK, RAS, EGFR, cyclin D1/3, MDM2, PTEN, CDK4, PDGFRA, PIK3CA, NF1, PIK3R1, LZTR1, BRAF, FGFR1, FGFR2, FGFR3, ATRX, TERT, NOTCH1, and FUBP1 [26]. The intra-tumor heterogeneity is currently vague in understanding and hence requires further research for the development of successful models for pre-clinical trials.

## 3. Diagnosis and imaging the brain tumor

Diagnosis of the brain tumor is primarily important for the identification and consideration of various drugs for the tumor. MRI is one of the standards for the diagnosis of glioblastoma. Properties, such as, the diffused rate of water, cerebral blood volume, blood flow rate, and transit time indicate the presence of tumor in the brain. The tracer 2-[18F] fluoro-2-deoxy-d-glucose (FDG) used in PET scan is ineffective in characterizing gliomas due to the presence of high levels of glucose in the brain tissues compared to the brain cells. An alternative to the positron emission tomography (PET) scan, the FET O-(2-[18F] fluoroethyl)-l-tyrosine has better resolution for brain imaging as the presence of massive levels of glucose does not permit PET scans effectively in brain tumors [27]. For detection of the Inter-tumor heterogeneity, MRI combined with NMR (nuclear magnetic resonance) spectroscopy indicated a positive approach in detecting of mesenchymal subtype of glioblastoma [28]. Magnetic resonance spectroscopy imaging (MRSI) is another commonly used technique that monitors the lactate, choline, creatine, N-acetyl aspartate and lipids in determining the tumor. The cellular levels measured by the MRSI indicate the tumor activity and it is a developing approach for the identification of various clones present in the tumor.

Fluorescent marker 5-aminolevulinic acid is a novel method in its phase 3 trials showing better surgical resection efficiency. The technique is a way of employing the marker 5-aminolevulinic which is taken up by the tumor cells, and fluorescence is observed under filtered light. The fluorescent dye is administered orally 2–5 h before the procedure showing maximal fluorescence at 6–8 h [29]. However, the fluorescent dye uptake does not occur in the normal brain tissue, blood vessels, and olfactory traces. The method is approved by the Food and Drug Administration (FDA) in the year 2017 [1] and it has shown promising potentials in improving the progression free survival (PFS). Fluorescence guided resection has greater scope in increasing the EOR (extent of resection) and survival in glioblastoma patients [30].

## 4. Conventional therapeutics for glioblastoma

The existing therapeutics for the treatment of glioblastoma are mainly dependent on surgical resection of the brain tumor. The surgery is subsequently followed by radiation therapy and chemotherapy. The surgical resection does not completely remove the tumor as the brain tumor often tends to infiltrate nearby adjacent normal brain cells’ parenchyma and it is unreliable to remove without affecting the normal cells of the brain [31]. Complete surgical resection in any case of brain tumor is not technically possible. Hence, the recurrence of the tumor from the remaining cells is inevitable. The EOR is important for the suppression of tumors [32].

However, EOR is observed to be restricted to a certain limit. For several decades, resection and radiation therapies were practiced in glioblastoma. Furthermore, there was no FDA-approved drug for treatment of glioblastoma until the year 2005 [33].

**Fig. 4:**
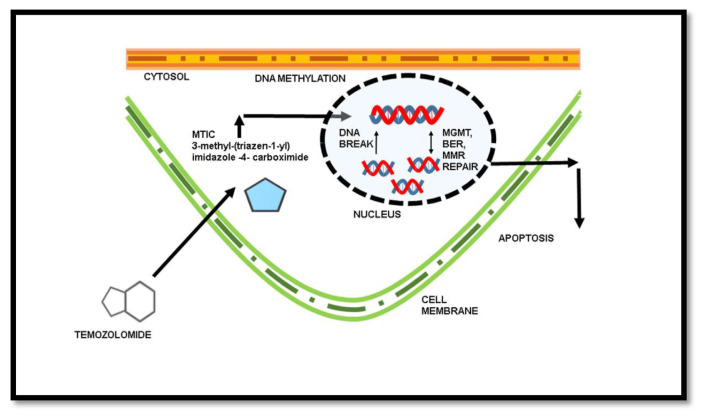
Mechanism of TMZ in the treatment of cancers by DNA methylation.

The drug temozolomide (TMZ) obtained Food and Drug Administration (FDA) approval for chemotherapy in glioblastoma in the year 2005. The implication of standard resection, radiotherapy and chemotherapy using TMZ has been in practice until recent advancements and drug approvals. The TMZ is a DNA alkylating agent and works in a way that causes the methylation of DNA in tumor cells [Fig. 4]. The methylation of DNA further promotes the cells to undergo apoptosis in the absence of MMR (mismatch repair), BER (base excision repair), and MGMT activity [34].

**Fig. 5:**
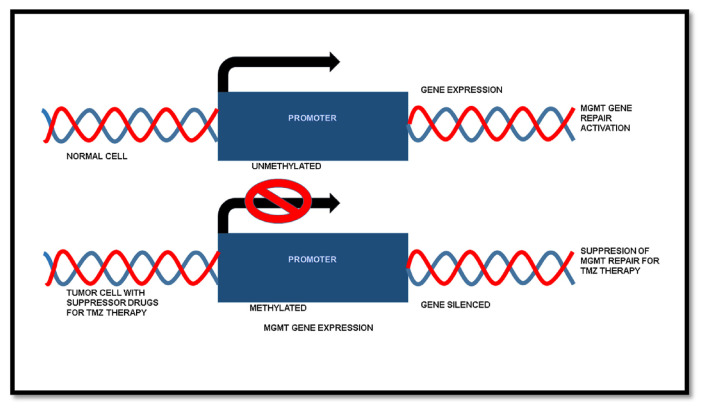
Silencing of MGMT promoter and gene expression as a Glioblastoma multiforme therapy for suppressing MGMT repair.

TMZ would have been a breakthrough in the treatment of glioblastoma, nonetheless, the MGMT (O-6-methylguanine DNA methyltransferase) gene in tumors is responsible for the repair of DNA, and hence nullifies the effect of TMZ [34]. In normal cells, the MGMT expression is regulated as a DNA repair mechanism, whereas, in tumor cells, the gene is silenced in certain profiles. The MGMT gene is mostly obtained as unmethylated in primary glioblastoma brain tumors, while in secondary glioblastoma brain tumors, the MGMT occurs as a methylated one. Therefore, the treatment with TMZ is found to be very effective in brain tumors with methylated MGMT promoter, i.e. in the secondary glioblastoma, as it plays a critical factor for the efficacy of TMZ [35]. MGMT promoter methylation acts as a biomarker that has a major significance in determining the prognosis of patients under treatment [36]. However, research to identify adjuvants with TMZ that causes the methylation of MGMT promoter is still in progress [Fig. 5]. The role of MGMT gene expression in the treatment of glioblastoma has a major significance. Screening of drugs for silencing the MGMT promoter might provide better treatment and prognosis in TMZ therapy [37]. Advancements in specific drug discoveries for targeting MGMT promoters, occurring in glioblastoma, can lead to better prognosis and OS in several cases with MGMT unmethylated tumor cells, i.e. in primary glioblastoma patients.

## 5. Advancements in precision medicine

The inquisitiveness in the area of research in precision medicine has bloomed over recent years with the advancement in precision medicine and breakthroughs in leukemia with drugs like “Gleevac” (Imatinib) acting as a signal transduction inhibitor [38]. The intense research has further led to the discovery of drugs and therapies, particularly for glioblastoma. Meanwhile, several drugs have been FDA approved and are under trials showing diverse results in patients with glioblastoma as shown in [Table 1]. The combination of CCNU (lomustine) along with temozolomide (TMZ) has shown effective and reliable results in patients with methylated MGMT promoter. All patients considered for the study had a Karnofsky performance score of >70 i.e., all patients were able to perform normal activity with few signs of symptoms and difficulties. The study observed a significant difference in median OS of patients and concluded the combined therapy to be better compared to the standard TMZ therapy. The research has further provided consistent results suggesting the use of combination therapy or personalized therapy, viz., both CCNU/temozolomide for newly diagnosed glioblastoma patients with methylated MGMT promoter being superior to the use of single therapy [39].

The use of monoclonal antibodies and tumor immunology-based therapies have also come to light. The brain cell’s exposure to immune cells such as the dendritic cells has shown tumor suppression [11,40]. Use of peptide vaccines for the EGFR mutation, a mutant tyrosine kinase, called as ACTIVATE, is in Phase II trials showing potential possibilities in clinical trials. Clinical trials showed positive results with increased survival to 26 months for glioblastoma patients [41].

For recurrent/post progression resection glioblastoma, it has been demonstrated that the monoclonal antibody Bevacizumab against VEGF displayed effective results with PFS for >6 months. Bevacizumab, approved in 2009, have also shown prospects in therapy against recurrent glioblastoma in combinations like CCNU/Bevacizumab [1].

BCNU Wafers (polycarboxyphenoxypropane/sebacic acid anhydride) containing nitrosourea carmustine also displays an effective delivery system through an implantable controlled – release approach. It is a technique by which the drug, approved by the FDA in 2003, is released to the site of the tumors through diffusion [42]. This system of implantable release of drugs has been shown to be effective for increasing PFS in glioblastoma.

Catheter-based convection-enhanced delivery (CED) is a system that employs the use of positive pressure infusion into the brain parenchyma with lower toxicity and effective delivery to a larger area of the tumor [44]. Real time tracking of the procedure can be done using intraoperative MRI. Drugs like Irinotecan and Imatinib can be effectively infused using the catheter-based CED. This type of CED delivery systems demonstrate effective advantages in targeting the tumor without promoting toxicity, and also enabling the ability to bypass the BBB [45]. Selective targeting of tumor cells enables both preclinical and clinical trials to be conducted with accuracy. Drug delivery systems developed over the years have proven to be reliable and efficient in cancer treatments.

Tyrosine kinase inhibitors (TKI) have also been used in trials for the inhibition of glioblastoma in most cases. Many TKIs showed negative outcomes and impacts in clinical trials in patients. The inability of TKIs to penetrate the BBB has also been a chief indication of its lower application in treating glioblastoma [46]. Few of the notable second generation TKI’s include neratinib, dacomitinib, afatinib, while the first-generation gefitinib, and the peptide vaccine rindopepimut (also known as CDX-110) have shown promising results so far. Use of multiple combinations of immunotherapies for targeting the over-expressive immunosuppression by PD-L1 (programmed cell death protein - ligand 1) and CTLA4 (cytotoxic T-lymphocyte-associated antigen 4) have effective potential in the suppression of glioblastoma [47]. Drugs having immunotherapeutic approaches also include nivolumab, durvalumab, DCVax-L, pembrolizumab, and tremelimumab, and are better known to target PD-L1 for effective immune response and therapy.

**Fig. 6:**
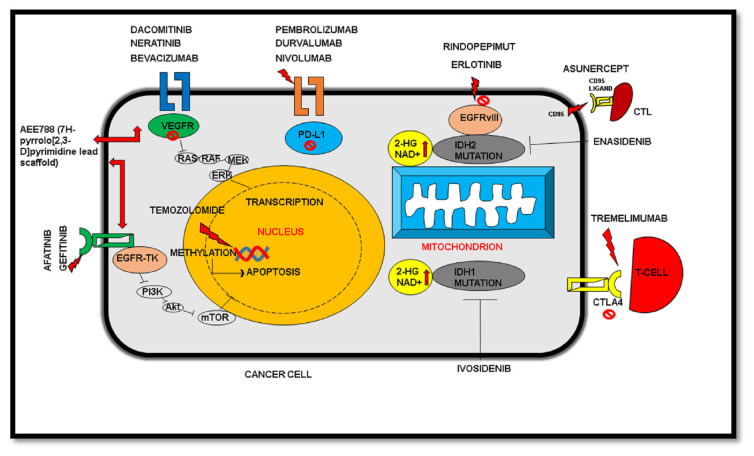
Mechanistic pathways for drugs and their targets under trial. PD-L1: Programmed cell death –ligand 1. EGFR: Epidermal growth factor receptor. VEGFR: Vascular endothelial growth factor receptor. IDH1 & IDH2: Isocitrate Dehydrogenase 1&2. TK: Tyrosine kinase. CTLA-4: Cytotoxic T Lymphocyte-associated antigen. CTL: Cytotoxic T Lymphocytes.

On the other hand, enasidenib and ivosidenib, act as inhibitors of IDH (isocitrate dehydrogenase), an essential enzyme of the TCA cycle. Detailed description of the mechanism of drugs on various targets have been illustrated below [Table 2 and Fig. 6]. However, evidences from existing reports reveal several constraints that are yet to be revised and redesigned for better prognosis in patients.

**Fig. 7:**
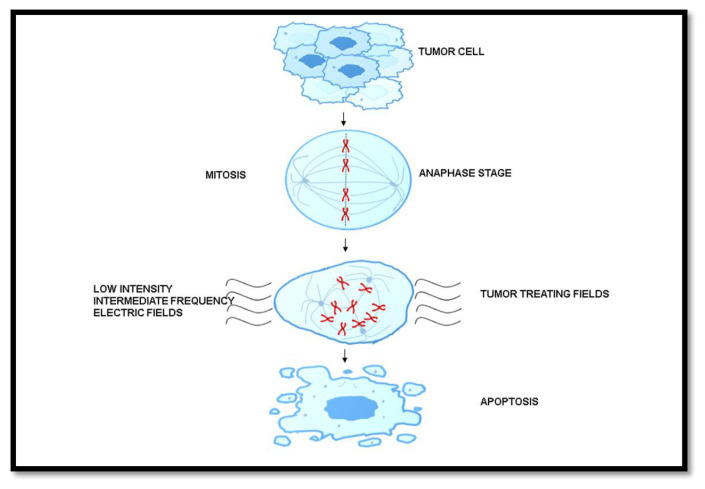
Mechanism of Tumor Treating Fields (TTF) during glioblastoma treatment. Disruption of cell at anaphase stage by inducing low intensity intermediate frequency electric fields are shown.

Tumor Treating Fields (TTF) is a technique of employing low-intensity intermediate frequency electric fields and applied on the patients’ shaved scalps [43]. Such types of treatments are antimitotic that target rapidly dividing cancer cells. These electric fields disrupt the microtubule stability, thereby disrupting mitotic division and causing cell deaths [Fig. 7]. Patients with newly diagnosed glioblastoma and those treated with TTF for 18 h per day together with adjuvant TMZ had a significantly improved OS from 16 months in all the TMZ group alone, and 20.9 months in the TTF plus TMZ combined groups. Such treatments resulted in the increased survivability of subjects by two-fold [49]. However, there are certain limitations to the TTF therapy, wherein the tedious treatment procedure applied on patients resulted in decline in their quality of life. Numerous patients are reported to discontinue the TTF therapy owing to its tedious process. Yet, TTF, amongst other therapies so far known, stands out from other treatments as it does not have side effects caused by chemotherapy [48]. Also, premature termination of the TTF treatment may have adverse effects on the patient and that may cause variations in survival efficiency [50].

## 6. Challenges in precision medicine

The most regarded barrier in the advancement of novel drugs and therapies has been the incapability of delivering drugs to the target tumor due to the presence of the BBB. Another major perspective on glioblastoma is the decline in the quality of life of patients as the cancer progresses. Poor lifestyle changes and therapy procedures inversely affect the well-being and survival of patients. This study has shown significant strategies to target the tumor by employing various methods. The advancement of drugs in the field of precision medicine for glioblastoma needs to be characterized among patients into specific subdivisions for identification and betterment of the treatment provided.

## 7. Conclusion

**Fig. 8:**
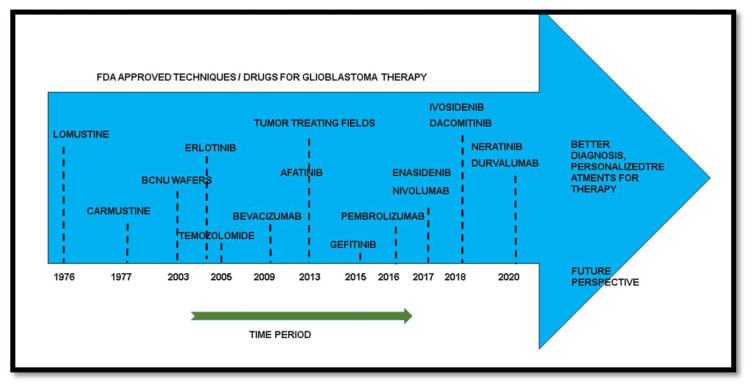
Food and Drug Administration (FDA) approved drugs over the period time for the treatment of glioblastoma.

It is evident that the conventional treatments of glioblastoma need alterations and are not efficient due to the malignancy and diversity of the tumor occurring among patients. GWS has shown the potential ability to treat the disease with information and studying of various genomic profiles. Targeting glioblastoma based on the genomic profile of the patient proves to be a viable way of approaching the tumor with potential therapeutic ability. The time period on discovery of drugs over the years has been represented in the illustration [Fig. 8]. Temozolomide, being a standard therapy for glioblastoma patients along with surgery and chemotherapy has evolved over the years into different drugs targeting a variety of pathways for better prognosis in patients. These drugs in different combinations show potential for targeting the patient’s tumor based on the profile.

The specific tumor characterization in patients using advanced diagnosis methods like FET (O-(2 [(18)F]fluoroethyl)-l-tyrosine) and MRSI (Magnetic resonance spectroscopic imaging) have showed a way for effective treatment using specific or different combination of drugs. Characterization of different models of glioblastoma based on the genomic profile is a better way to hypothesize therapeutic outcomes. Therapeutics by means of converging tumor characteristics and drug mechanisms may show potential in tumor treatment. The future perspective of personalized medicine in glioblastoma is inclined towards a novel method of treatment with precise combination of drugs. Many drugs are under trials and possess potentiality to treat patients with glioblastoma by specific and multiple therapies. The attitude of adhering to conventional methods of treatment using surgery and radioactive therapies by the scientific and medicinal community due to ethical reasons has been a minor limitation for the dawdling type progress of precision medicinal therapeutics. This study has provided significant therapeutic approaches for a better prognosis of glioblastoma. The evolution in the field of GWS and both availability and unrestricted access of medicinal advancements throughout the communities can demonstrate development in the field. Early prediction and diagnosis using genomic markers can display major significances in the treatment and lifestyle of individuals.

Advancements and findings in therapies using precision medicine approach for cancer treatments especially in the field of glioblastoma is inevitable. Expansion of the study can improve prognosis and the current situation of glioblastoma in patients. Further research and outcomes from the scientific community through precision medicine promises a breakthrough in the treatment of glioblastoma.

## Figures and Tables

**Table 1:** FDA-approved drugs for treatment of glioblastoma - a high grade glioma (HGG).

Sl No.	Drug/Therapy employed	FDA approval and year	Mechanism	Approved for	Application Route	OS	PFS @6months duration
1.	TMZ (Temozolomide)	FDA approval in 2005	Nonspecific alkylating agent that causes mismatch repair in DNA by methylation at the O6 position of guanine	All high grades of glioma	Oral	14.616.1 months	53.90% [1]
2.	Lomustine (CCNU)	FDA approval in 1976	Nonspecific alkylating agent that causes crosslinking of DNA and RNA in dividing cells triggering cell death	Recurrent HGG (High Grade Glioma)	Oral	11.5 months	No supporting data yet [42]
3.	Carmustine (BCNU)	FDA approval in 1977	Nonspecific alkylating agent that causes crosslinking of DNA and RNA in dividing cells; also binds to and modifies glutathione reductase	Recurrent HGG	IV	11.75 months	No supporting data yet [42]
4.	Carmustine wafer implants (BCNU wafers)	FDA approval in 2003	Nonspecific alkylating agent that causes crosslinking of DNA and RNA in dividing cells; also binds to and modifies glutathione reductase	Recurrent HGG	Implanted	13.9 months	No supporting data yet [42]
5.	Bevacizumab (BVZ)	FDA approval in 2009	Targeted therapeutic antibody that binds and inhibits VEGF protein in tumor cells	Recurrent HGG	IV	9.3 months	36% [1]
6.	TTF (Tumor treating Fields)	FDA approval in 2015	Low-intensity (1–3 V/cm), intermediate frequency (200 kHz) alternating electric fields that disrupt mitosis in tumor cells	Recurrent and new HGG	On scalp	20.520.9 months	56% [43]

**Table 2:** Recent advancements in agents available for future therapies.

Sl No.	Agent	Mechanism	Approval/Clinical/Pre-clinical stage	Administration	Grade	Results
1.	Nivolumab (Opdivo)	Nivolumab is an immunoglobulin that inhibits PD-1 antibody.	Phase III	IV	Recurrent and new gliomas	Median OS 9.5 months [40]
2.	Gefitinib	Targets the EGFR TKI	Phase II	Oral	Recurrent GBM	No supporting data yet [42]
3.	Durvalumab	It is an immunoglobulin G1 kappa monoclonal antibody that inhibits the PD-1, PD-2 and CD80.	Phase II	IV	Recurrent and new GBM	OS 59% and PFS 50% [44]
4.	DCVax-L	Use of patients’ dendritic cells for inhibiting recurrence of GBM	Phase II	Direc t	Recurrent GBM alone	>12 months with 93.5% efficiency [40]
5.	Afatinib	Targets the EGFR and EGFRv III (deletion of exons 2–7) TK1	Phase I, II	Oral	Recurrent GBM	No supporting data yet [41]
6.	Tremelimumab and Durvalumab	Targets the PD-1 and CTLA-4 by inhibition	Phase II	IV	All and recurrent HGG	No supporting data yet. Clinical Data Identifier NCT02794883 [47]
7.	AEE788 (7Hpyrrolo [2,3-D] pyrimidine lead scaffold)	EGFR (epidermal growth factor receptor)/VEGFR (vascular endothelial growth factor receptor) TKI	Phase I	Oral	Recurrent GBM	No supporting data/study discontinued [48]
8.	Dacomitinib (a Pan-HER irreversible inhibitor)	EGFR TKI 2nd generation	Phase II	Oral	Recurrent GBM	NCT01520870 (PF-299804) [46]
9.	Neratinib	EGFR TKI 2nd generation	Phase II	Oral	Recurrent GBM	NCT01953926 (HER mutation study [46]
10.	Nivolumab + Bevacizumab	Inhibits PD-1 antibody and VEGF protein in tumor cells.	Phase III	IV	Recurrent GBM	10 months OS [47]
11.	Pembrolizumab	Blocks the protein PD1	Phase II	Oral	Recurrent GBM	PFS at 6 months [47]
12.	Enasidenib	Allosteric inhibiter of mutant IDH2	Phase II	Oral	All types of gliomas	No supporting data [46]
13.	Ivosidenib	Small molecular inhibitor for mutant IDH1	Phase I	Oral	All types of gliomas	PFS 13 months [46]
14.	Erlotinib	Targets EGFRvIII (deletion of exons 2–7) TKI	Phase II	Oral	Recurrent glioma	OS at 12 months, PFS at 6 months [41]
15.	Rindopepimut (EGFRvIII peptide vaccine)	Targets EGFRvIII mutation	Phase III	Oral	Newly diagnosed GBM	OS at 20.1 months. Ineffective & discontinued after Phase III [41]
16.	Asunercept	Targets CD95/CD95 ligand & Blocks CD95 ligand	Phase II	IV	Recurrent GBM	PFS at 11.2–33.4 for rRT + APG101(Asune rcept) [42]
17.	Depatux-M (depatuxizumab mafodotin)	EGFR antibody drug conjugate, release of anti-microtubule agent	Phase III	IV	Newly diagnosed GBM	Ineffective results, study discontinued [42]
